# Effectiveness of Amenamevir Vs. Valacyclovir in Preventing Herpes Zoster‐Related Hospitalization and Complications: A Target Trial Emulation Using a Large Japanese Claims Database

**DOI:** 10.1111/1346-8138.70349

**Published:** 2026-06-09

**Authors:** Yasuhiro Kano, Yuya Kimura, Hideo Yasunaga

**Affiliations:** ^1^ Department of Clinical Epidemiology and Health Economics, School of Public Health The University of Tokyo Tokyo Japan; ^2^ Department of Emergency and General Medicine Tokyo Metropolitan Tama Medical Center Tokyo Japan; ^3^ Department of Health Services Research, Graduate School of Medicine University of Tokyo Tokyo Japan

## Abstract

Amenamevir is a novel anti‐herpes zoster drug that is increasingly used in Japan because of its once‐daily administration and lack of renal dose adjustment requirements. However, evidence of its clinical effectiveness is limited. This study aimed to estimate the clinical effectiveness of amenamevir vs. valacyclovir in preventing zoster‐related hospitalizations and complications. Using a nationally representative claims database from 2017 to 2024, we conducted a retrospective cohort study with a new‐user, active‐comparator design. We included adults (aged ≥ 18 years) with uncomplicated herpes zoster who initiated amenamevir or valacyclovir treatment. Propensity score overlap weighting was used to adjust for differences between patients prescribed amenamevir and valacyclovir. The primary outcome was 30‐day zoster‐related hospitalization. The secondary outcomes included diagnoses of zoster‐related complications (zoster encephalitis, zoster meningitis, disseminated zoster, and zoster ocular disease) and acute kidney injury. In total, 67 912 individuals (38.2% male; mean age, 70.1 years) were included. After overlap weighting, baseline covariates were well‐balanced between the groups. The risk of 30‐day zoster‐related hospitalization was similar between amenamevir and valacyclovir users (0.73% vs. 0.73%; risk ratio [RR], 1.00; 95% confidence interval [CI], 0.84–1.20). For secondary outcomes, zoster encephalitis (0.02% vs. 0.01%; RR, 2.09; 95% CI, 0.62–7.10), zoster meningitis (0.04% vs. 0.02%; RR, 2.32; 95% CI, 0.89–6.09), and disseminated zoster (0.13% vs. 0.10%; RR, 1.25; 95% CI, 0.79–1.97) did not differ significantly between the groups. However, amenamevir was associated with a higher risk of zoster ocular disease (0.84% vs. 0.67%; RR, 1.25; 95% CI, 1.05–1.49) and a lower risk of acute kidney injury (0.02% vs. 0.09%; RR, 0.23; 95% CI, 0.10–0.53). Based on our findings, amenamevir may be a useful option for herpes zoster, particularly in clinical settings where timely assessment of renal function is not available. However, caution is warranted regarding possible increases in ocular complications.

## 1 Introduction

Herpes zoster is common worldwide, affecting approximately one in three individuals over their lifetime [1]. Complications of herpes zoster, including encephalitis, meningitis, disseminated zoster, and zoster ocular disease, can cause persistent sequelae, impair quality of life, and be fatal in rare cases. Antiviral therapy reduces the risk of these complications [2, 3, 4].

Although oral valacyclovir is a standard therapy for uncomplicated herpes zoster, its need for renal dose adjustment, multiple daily dosing, and potential nephrotoxicity may limit its use, especially when the assessment of renal function is not immediately available. Amenamevir, a helicase‐primase inhibitor, is a novel anti‐herpes zoster agent approved in Japan in 2017 [5]. Amenamevir is advantageous in that it does not usually require dose adjustment based on renal function because it is mainly eliminated in feces [6], and it can be administered once daily because of its long half‐life. Owing to this convenience of prescription, amenamevir has become commonly used for uncomplicated herpes zoster in Japan [7].

However, clinical evidence of the effectiveness of amenamevir remains limited. Although a randomized, double‐blind, phase 3 trial showed the non‐inferiority of amenamevir to valacyclovir [8], its generalizability is limited. The trial was restricted to a predominantly non‐older adult, immunocompetent population and had a small sample size [8]. In addition, the primary endpoint was the proportion of cessation of new lesion formation by day 4, which does not adequately assess the prevention of zoster‐related complications. Furthermore, a theoretical concern exists that amenamevir may increase the risk of zoster‐related complications [7]. A mouse study showed that the cerebrospinal fluid‐to‐plasma concentration of amenamevir was approximately one‐tenth [9], which may be lower than that of acyclovir and its prodrug, valacyclovir [10, 11, 12]. Several case reports and small observational studies have described central nervous system (CNS) involvement developing after amenamevir treatment in patients with herpes zoster [7, 13, 14, 15, 16]. These issues underscore the need for large‐scale studies to evaluate the effectiveness of amenamevir [17].

To address this evidence gap, we conducted a new‐user, active‐comparator cohort study emulating a target trial of amenamevir vs. valacyclovir to compare the risk of treatment failure in herpes zoster using a nationwide administrative claims database in Japan.

## 2 Methods

### 2.1 Data Source

We used the DeSC database, a commercially available administrative claims database in Japan. The DeSC database includes data on approximately 13.5 million individuals and has been validated as representative of the Japanese population in terms of age, sex, and the prevalence of common comorbidities [18]. The database contains information on patient characteristics, diagnoses, procedures, and dispensed prescriptions. It includes annual health checkup data covering approximately 30% of the registered population [19]. This study was approved by the Institutional Review Board of the University of Tokyo (2021010NI, April 23, 2021). The requirement for informed consent was waived because of the anonymous nature of the data.

### 2.2 Emulation of the Target Trial

We designed this study using a target trial emulation framework [20, 21], Details of the protocol for the hypothetical pragmatic randomized trial and the emulation procedure are provided in Table S1. The hypothetical target trial was a pragmatic randomized controlled trial comparing amenamevir and valacyclovir in patients with uncomplicated herpes zoster.

### 2.3 Study Population

We included patients aged ≥ 18 years who had an outpatient diagnosis of herpes zoster and received amenamevir or valacyclovir on the same day between September 7, 2017 (the release date of amenamevir in Japan), and July 31, 2024. The index date was defined as the date of herpes zoster diagnosis and prescription of amenamevir or valacyclovir. The detailed study design is shown in Figure S1. We excluded patients who did not have at least 365 days of continuous enrollment before the index date and those who lacked baseline estimated glomerular filtration rate (eGFR) data from annual health checkups. To emulate a new‐user design, we excluded patients with any prior diagnosis of herpes zoster or prior use of amenamevir or valacyclovir during the 365‐day washout period. We excluded patients who were hospitalized on the index date; had coexistent varicella; had herpes zoster‐related complications (including encephalitis, meningitis, disseminated zoster, or ocular involvement); received prescriptions for two or more varicella‐zoster virus‐active antivirals (amenamevir, valacyclovir, acyclovir, vidarabine, or famciclovir) on the index date; were receiving rifampicin (a contraindication to amenamevir); were undergoing dialysis; were pregnant; or had human immunodeficiency virus infection. Detailed definitions of the inclusion and exclusion criteria are provided in Table S2. If a patient had two or more eligible episodes during the study period, only the first episode was included in the analysis.

### 2.4 Outcomes

The primary outcome was 30‐day zoster‐related hospitalization, defined as hospitalization with intravenous antiviral therapy (acyclovir or vidarabine) on the day of hospitalization or the following day within 30 days after the index date. We assumed that these hospitalizations represented admissions for the treatment of acute zoster‐related complications because the database did not consistently contain information on the principal diagnosis at admission. This operational definition was consistent with the current Japanese clinical practice guidelines for herpes zoster [22], which recommend reserving inpatient intravenous therapy for patients with suspected complications or high risk of severe disease. Patients were followed up from the index date to the earliest outcome of interest: death, loss of insurance eligibility (end of observable data), or 30 days of follow‐up. Secondary outcomes, which were assessed using the same follow‐up scheme, included zoster‐related complications (zoster encephalitis, zoster meningitis, disseminated zoster, and zoster ocular disease) and acute kidney injury (AKI). AKI was defined according to the corresponding International Classification of Diseases, Tenth Revision, or dialysis procedure codes. Detailed definitions of the outcomes are provided in Table S3.

### 2.5 Covariates

We collected covariate information during the 365 days before the index date or on the index date, as appropriate. Baseline characteristics included age; sex; distribution of herpes zoster (craniocervical vs. non‐craniocervical); most recent eGFR from annual health checkup data; comorbidities; Charlson Comorbidity Index; medication use (including nonsteroidal anti‐inflammatory drugs, systemic glucocorticoids, antineoplastic agents, and immunosuppressants); type of treatment facility (clinic, hospital, or others); healthcare utilization (annual number of outpatient visits and any hospitalization in the prior year); health insurance plan type; and fiscal year of the index date. The comorbidities included hypertension, dyslipidemia, diabetes mellitus, chronic obstructive pulmonary disease, congestive heart failure, coronary artery disease, stroke, immune system disorders, solid tumors, hematological malignancies, other hematological diseases with immunocompromised conditions, and organ transplantation. These variables were selected a priori based on clinical relevance and previous literature [17, 23, 24, 25, 26]. Detailed definitions of covariates are provided in Table S4.

### 2.6 Statistical Analyses

Propensity score overlap weighting was used to balance the measured confounders between the two groups. First, we estimated the propensity scores for amenamevir (vs. valacyclovir) using a multivariate logistic regression model that included all baseline covariates as independent variables. No missing data were reported for the variables used in the primary analysis. Overlap weights were assigned as 1—propensity score for patients treated with amenamevir and propensity score for those treated with valacyclovir; as a result, the weighted sample represented the population in whom treatment choice was most uncertain [27]. Overlap weighting optimizes the precision of treatment‐outcome estimates among a broad class of propensity score weighting methods, including inverse probability of treatment weighting and matching analogs [27]. After weighting, we assessed covariate balance between treatment groups using standardized mean differences, with an absolute value < 0.10, indicating adequate balance. For the primary and secondary outcomes, we fitted weighted modified Poisson regression models with a log link and robust (sandwich) variance estimators to obtain risk ratios (RRs) and 95% confidence intervals (CIs) for amenamevir compared with valacyclovir. Absolute risks in each treatment group were estimated from the fitted model, and risk differences (RDs) were calculated as the risk in the amenamevir group minus the risk in the valacyclovir group. The CIs for the RDs were derived using the delta method, based on the robust covariance matrix. All analyses were performed according to the antiviral agent dispensed on the index date; i.e., patients were analyzed in the groups initially assigned at treatment initiation (observational analog of intention‐to‐treat).

### 2.7 Subgroup and Sensitivity Analyses

We performed subgroup analyses according to age (< 80 vs. ≥ 80 years), baseline eGFR (≥ 60, 45–59, and ≤ 44 mL/min/1.73 m^2^), and distribution of zoster (craniocervical vs. non‐craniocervical lesion). Subgroup analyses were performed to assess the effectiveness of amenamevir in patients aged ≥ 80 years who were excluded from a previous randomized controlled trial [8], patients with chronic kidney disease, and patients at high risk of zoster‐related complications.

We performed a sensitivity analysis to assess the robustness of the primary findings. First, we re‐estimated the treatment effect using propensity score matching and conventional stabilized inverse probability of treatment weighting instead of overlap weighting. Second, to increase the specificity of the primary outcome definition used in the primary analysis, we required a diagnostic code for herpes zoster‐related complications recorded on the day of admission. We used R version 3.6.3 (R Foundation for Statistical Computing, Vienna, Austria) for all statistical analyses.

## 3 Results

### 3.1 Patient Characteristics

**FIGURE 1:**
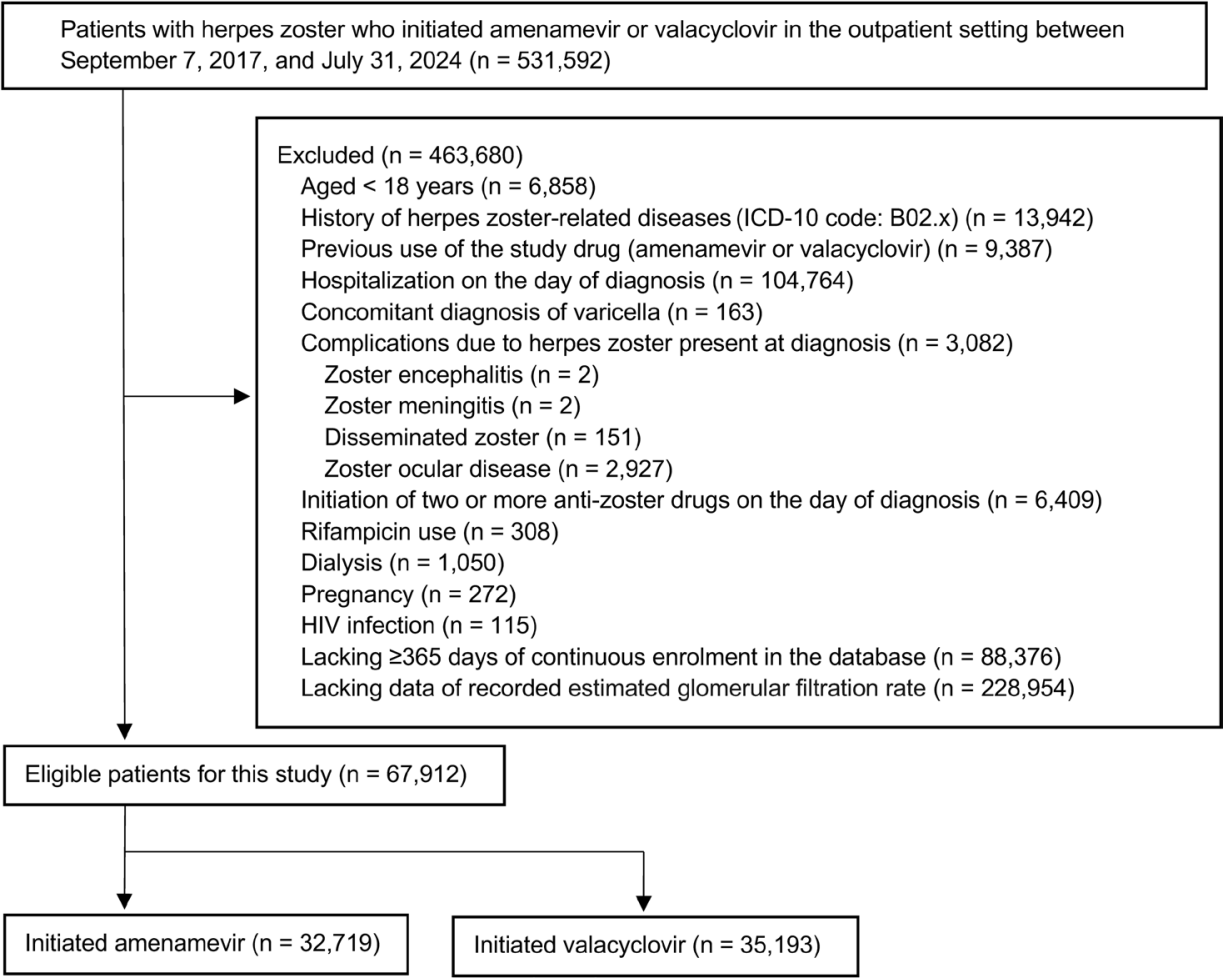
Study flowchart.

We identified 531 592 patients who received amenamevir or valacyclovir for the treatment of zoster during the study period. After applying the exclusion criteria, 67 912 patients were included in this study (Figure 1). Overall, the mean age was 70.1 years, and 38.2% of the patients were male. Table 1 shows the baseline patient characteristics of the amenamevir and valacyclovir groups before and after propensity score overlap weighting. Before weighting, the amenamevir group was older by two years (mean) and had more comorbidities than the valacyclovir group. Amenamevir use increased across the fiscal years and exceeded valacyclovir use from 2022 onward. The distribution of treatment facility types (clinic or hospital) was similar between the two groups. After propensity score overlap weighting, all baseline characteristics were well‐balanced between the groups.

**TABLE 1:** Cohort characteristics before and after propensity score overlap weighting.

	Before weighting			After weighting		
	Amenamevir	Valacyclovir		Amenamevir	Valacyclovir	
Variables	*n* = 32 719	*n* = 35 193	ASD	*n* = 16 668^a^	*n* = 16 668^a^	ASD
Age, years, mean (SD)	71.3 (11.0)	69.0 (12.0)	0.194	70.3 (11.5)	70.3 (11.4)	< 0.001
Male sex, *n* (%)	12 548 (38.4)	13 423 (38.1)	0.004	6 353 (38.1)	6 353 (38.1)	< 0.001
Craniocervical zoster, *n* (%)	1 318 (4.0)	1 239 (3.5)	0.027	627 (3.8)	627 (3.8)	< 0.001
Baseline eGFR (mL/min/1.73 m^2^), mean (SD)	67.1 (14.9)	68.8 (15.4)	0.108	67.9 (15.1)	67.9 (14.7)	< 0.001
Comorbidities, *n* (%)						< 0.001
Hypertension	16 626 (50.8)	16 903 (48.0)	0.056	8 263 (49.6)	8 263 (49.6)	< 0.001
Dyslipidemia	16 141 (49.3)	16 900 (48.0)	0.026	8 138 (48.8)	8 138 (48.8)	< 0.001
Diabetes mellitus	8 339 (25.5)	8 327 (23.7)	0.042	4 106 (24.6)	4 106 (24.6)	< 0.001
COPD	769 (2.4)	720 (2.0)	0.021	368 (2.2)	368 (2.2)	< 0.001
Congestive heart failure	3 660 (11.2)	3 286 (9.3)	0.061	1 706 (10.2)	1 706 (10.2)	< 0.001
Coronary artery disease	3 781 (11.6)	3 827 (10.9)	0.022	1 876 (11.3)	1 876 (11.3)	< 0.001
Stroke	1 859 (5.7)	1 874 (5.3)	0.016	922 (5.5)	922 (5.5)	< 0.001
Immune system disorders	91 (0.3)	76 (0.2)	0.013	41 (0.2)	41 (0.2)	< 0.001
Solid tumor	3 114 (9.5)	2 925 (8.3)	0.042	1 487 (8.9)	1 487 (8.9)	< 0.001
Hematological malignancies	198 (0.6)	216 (0.6)	0.001	102 (0.6)	102 (0.6)	< 0.001
Other hematological diseases with immunocompromised conditions	191 (0.6)	181 (0.5)	0.009	92 (0.5)	92 (0.5)	< 0.001
Organ transplants	19 (0.1)	17 (0.0)	0.004	9 (0.1)	9 (0.1)	< 0.001
Charlson Comorbidity Index, mean (SD)	1.22 (1.56)	1.11 (1.47)	0.074	1.17 (1.52)	1.17 (1.51)	< 0.001
NSAID prescription, *n* (%)	14 183 (43.3)	15 406 (43.8)	0.009	7 259 (43.5)	7 259 (43.5)	< 0.001
Immunosuppressant prescription, *n* (%)	493 (1.5)	508 (1.4)	0.005	248 (1.5)	248 (1.5)	< 0.001
Systemic glucocorticoid prescription, *n* (%)	1 447 (4.4)	1 423 (4.0)	0.019	703 (4.2)	703 (4.2)	< 0.001
Antineoplastic agent prescription, *n* (%)	231 (0.7)	230 (0.7)	0.006	113 (0.7)	113 (0.7)	< 0.001
Treatment facility type, *n* (%)			0.022			< 0.001
Clinic	28 471 (87.0)	30 678 (87.2)		14 508 (87.0)	14 508 (87.0)	
Hospital	4 214 (12.9)	4 499 (12.8)		2 149 (12.9)	2 149 (12.9)	
Others^b^	34 (0.1)	16 (0.0)		10 (0.1)	10 (0.1)	
Number of outpatient visits per year, *n* (%)			0.072			< 0.001
≤ 11	10 992 (33.6)	12 952 (36.8)		5 838 (35.0)	5 838 (35.0)	
12–23	11 249 (34.4)	11 881 (33.8)		5 691 (34.1)	5 691 (34.1)	
≥ 24	10 478 (32.0)	10 360 (29.4)		5 139 (30.8)	5 139 (30.8)	
Hospitalization during the last 1 year, *n* (%)	1 674 (5.1)	1 515 (4.3)	0.038	784 (4.7)	784 (4.7)	< 0.001
Health insurance plan type			0.158			< 0.001
National Health Insurance (*Kokuho*)	3 071 (9.4)	4 481 (12.7)		1 795 (10.8)	1 795 (10.8)	
Health Insurance Societies (*Kempo*)	12 628 (38.6)	11 239 (31.9)		5 894 (35.4)	5 894 (35.4)	
Advanced Elderly Medical Service System	17 020 (52.0)	19 473 (55.3)		8 979 (53.9)	8 979 (53.9)	
Fiscal year of index date, *n* (%)			0.181			< 0.001
2017	17 (0.1)	58 (0.2)		13 (0.1)	13 (0.1)	
2018	1 628 (5.0)	2 445 (6.9)		971 (5.8)	971 (5.8)	
2019	5 207 (15.9)	7 015 (19.9)		2 967 (17.8)	2 967 (17.8)	
2020	5 866 (17.9)	6 974 (19.8)		3 162 (19.0)	3 162 (19.0)	
2021	7 601 (23.2)	7 670 (21.8)		3 778 (22.7)	3 778 (22.7)	
2022	7 528 (23.0)	6 804 (19.3)		3 546 (21.3)	3 546 (21.3)	
2023	4 424 (13.5)	3 753 (10.7)		2 005 (12.0)	2 005 (12.0)	
2024	448 (1.4)	474 (1.3)		227 (1.4)	227 (1.4)	

Abbreviations: ASD, absolute standardized mean difference; COPD, chronic obstructive pulmonary disease; eGFR, estimated glomerular filtration rate; NSAID, nonsteroidal anti‐inflammatory drug; SD, standard deviation.

^a^ Weighted number of patients.

^b^ “Others” includes dental hospitals, dental clinics, and other medical institutions.

### 3.2 Main Analysis

After overlap weighting, the 30‐day risk of zoster‐related hospitalization was similar between the amenamevir (0.73%) and valacyclovir (0.73%) groups (RR, 1.00; 95% CI, 0.84–1.20; RD, 0.00%; 95% CI, −0.13%–0.13%) (Table 2). The diagnoses of zoster encephalitis (0.02% vs. 0.01%; RR, 2.09; 95% CI, 0.62–7.10), zoster meningitis (0.04% vs. 0.02%; RR, 2.32; 95% CI, 0.89–6.09), and disseminated zoster (0.13% vs. 0.10%; RR, 1.25; 95% CI, 0.79–1.97) were not significantly different between the two groups. However, zoster ocular disease was significantly more frequent in the amenamevir group than in the valacyclovir group (0.84% vs. 0.67%; RR, 1.25; 95% CI, 1.05–1.49; RD, 0.17%; 95% CI, 0.04%–0.30%). AKI was less frequent in the amenamevir group than in the valacyclovir group (0.02% vs. 0.09%; RR, 0.23; 95% CI, 0.10–0.53; RD, −0.07%; 95% CI, −0.10% to −0.03%).

**TABLE 2:** Thirty‐day estimated risk for outcomes comparing amenamevir and valacyclovir.

Outcomes	Amenamevir	Valacyclovir	RR (95% CI)	RD (95% CI)
Primary outcome (%)				
30‐day zoster‐related hospitalization	0.73% (0.76%)	0.73% (0.71%)	1.00 (0.84–1.20)	0.00% (−0.13% to 0.13%)
Secondary outcomes (%)				
Diagnosis of zoster encephalitis	0.02% (0.02%)	0.01% (0.01%)	2.09 (0.62–7.10)	0.01% (−0.01% to 0.03%)
Diagnosis of zoster meningitis	0.04% (0.04%)	0.02% (0.02%)	2.32 (0.89–6.09)	0.02% (−0.00% to 0.05%)
Diagnosis of disseminated zoster	0.13% (0.13%)	0.10% (0.09%)	1.25 (0.79–1.97)	0.03% (−0.03% to 0.08%)
Diagnosis of zoster ocular disease	0.84% (0.86%)	0.67% (0.66%)	1.25 (1.05–1.49)	0.17% (0.04% to 0.30%)
Diagnosis of AKI or initiation of dialysis	0.02% (0.02%)	0.09% (0.08%)	0.23 (0.10–0.53)	−0.07% (−0.10% to −0.03%)

*Note:* Values in parentheses represent the crude outcomes. RR was calculated as the risk in the amenamevir group/risk in the valacyclovir group, and RD as the risk in the amenamevir group – risk in the valacyclovir group. RR > 1 and RD > 0 indicated a higher risk of amenamevir use.

Abbreviations: AKI, acute kidney injury; CI, confidence interval; RD, risk difference; RR, risk ratio.

### 3.3 Subgroup and Sensitivity Analyses

**FIGURE 2:**
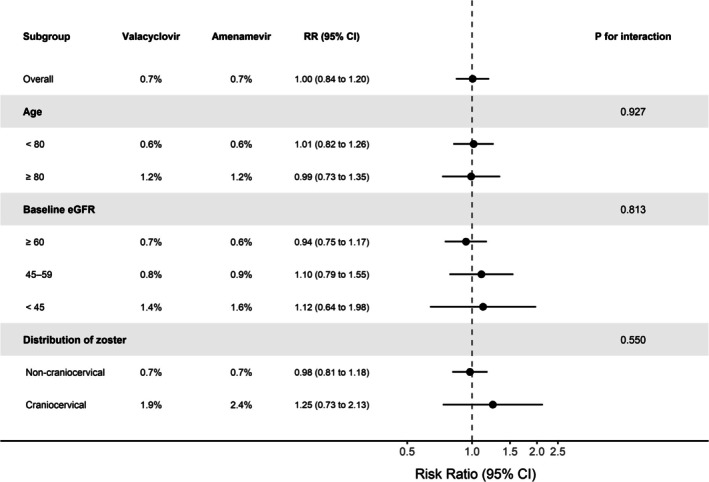
Subgroup analysis for 30‐day zoster‐related hospitalization between the propensity score weighted groups. eGFR, estimated glomerular filtration rate; RR, risk ratio; CI, confidence interval.

The results of the subgroup analysis are shown in Figure 2. Across all three subgroup dimensions (< 80 vs. ≥ 80 years, baseline eGFR of ≥ 60, 45–59, and ≤ 44 mL/min/1.73 m^2^, and craniocervical vs. non‐craniocervical lesion), the results were consistent with those of the main analysis, with no significant difference between the two groups. Sensitivity analyses using propensity score matching and inverse probability of treatment weighting, instead of overlap weighting, were consistent with the main analysis (Tables S5 and S6). An analysis using a more specific primary outcome definition, adding a diagnostic code for herpes zoster‐related complications on the admission date to the primary analysis definition, yielded consistent results (Table S7).

## 4 Discussion

To the best of our knowledge, this is the first real‐world study comparing the clinical outcomes of amenamevir and valacyclovir in patients with herpes zoster. Overall, 0.7% of the cohort experienced zoster‐related hospitalization (defined as an admission requiring intravenous antiviral therapy within 30 days). No significant difference was observed in zoster‐related hospitalization between the two groups, whereas amenamevir was associated with a higher risk of ocular zoster and a lower risk of AKI than valacyclovir.

Valacyclovir reduces zoster‐related hospitalizations and neurological complications [3, 4], whereas comparable clinical evidence for amenamevir is scarce. Even in patients with herpes zoster who present without CNS symptoms, subclinical viral inflammation in the CNS is common [28], and clinically overt CNS involvement can develop later, typically within 1 month [24, 29]. Although lower cerebrospinal fluid‐to‐plasma concentration of amenamevir has raised concerns about greater CNS involvement and subsequent hospitalization [7, 13, 14, 15, 16], our study observed no significant difference in zoster‐related hospitalization. Although the point estimate for encephalitis and meningitis in the amenamevir group was approximately two‐fold higher than that in the valacyclovir group, these events were rare (< 0.05%), resulting in wide 95% CIs and no significant differences. Therefore, although wide confidence intervals preclude a definitive conclusion on the risk of CNS complications with amenamevir use, the incidence of severe cases requiring hospital admission was comparable between the groups, with relatively narrow confidence intervals. Greater in vitro anti‐varicella‐zoster virus potency and once‐daily dosing of amenamevir, which may improve patient adherence and facilitate treatment completion, may partly explain these findings [30].

The risk of ocular complications was higher in the amenamevir group than in the valacyclovir group. Although the mechanism is unclear, differences in the ocular pharmacokinetics and/or pharmacodynamics between amenamevir and valacyclovir may be a possible hypothesis, including the potentially less effective suppression of varicella‐zoster virus replication within the trigeminal distribution. However, this remains speculative because comparative basic data on ocular pharmacokinetics are limited [9, 31, 32]. Based on these results, caution is warranted when prescribing amenamevir to patients at high risk of ocular complications (e.g., trigeminal herpes zoster with a positive Hutchinson's sign) [33]. Until this issue is further investigated, clinicians may consider prioritizing valacyclovir over amenamevir for these patients or ensuring close ophthalmologic follow‐up when amenamevir is used.

AKI, defined by either a diagnostic code for AKI or dialysis initiation, was significantly more frequent in the valacyclovir group than in the amenamevir group. As the diagnostic codes for AKI have high specificity but low sensitivity [34], our absolute risk estimates likely underestimated the true incidence. Furthermore, these findings may partly reflect surveillance (or detection) bias. As valacyclovir is a well‐recognized cause of crystal‐induced AKI, clinicians should monitor kidney function more closely in patients receiving valacyclovir. Although a more favorable renal safety profile for amenamevir may be biologically plausible, given its predominant fecal elimination, further studies incorporating AKI definitions based on laboratory testing results and/or measures of testing frequency to mitigate surveillance bias are warranted. Nevertheless, the lower observed risk of AKI may support a renal safety advantage of amenamevir, particularly in older patients or those at risk of renal impairment.

Based on our findings, amenamevir may be a valuable treatment option for herpes zoster. However, several clinical concerns warrant further investigation. First, compared with acyclovir and valacyclovir, amenamevir has less post‐marketing experience than acyclovir, and previously unrecognized adverse effects may be recognized as post‐marketing exposure increases. Second, as the use of amenamevir increases, varicella‐zoster virus strains carrying mutations that confer resistance to this novel antiviral agent may emerge. Given its potential role in the treatment of acyclovir‐resistant varicella‐zoster virus [35], some may argue that new antiviral drugs should be reserved whenever possible. Third, amenamevir is currently more expensive than valacyclovir; therefore, cost‐effectiveness analyses are warranted.

This study has some limitations. First, residual confounding factors may remain despite our attempt to adjust comprehensively based on clinical knowledge and prior studies. Therefore, some clinical information may have been incompletely captured. For example, craniocervical involvement, a known risk factor for complications, accounts for approximately 10%–20% of patients with herpes zoster [36, 37], whereas only 3.8% of the cases in our study were classified as craniocervical. We were unable to obtain vaccination records; therefore, residual confounding due to zoster vaccination status could not be excluded. However, clinicians may be unlikely to differentially select amenamevir vs. valacyclovir based on a patient's vaccination history. Second, requiring baseline eGFR data may have introduced selection bias, because individuals with available health checkup data are likely to represent a relatively healthier population. Baseline eGFR data were required because preexisting renal impairment is considered an important potential confounder. Nevertheless, the generalizability of our results may be limited because the study population may represent a relatively healthier subset of patients than those observed in routine clinical practice. Finally, additional evidence is required to confirm the reproducibility of our findings in healthcare settings outside Japan.

In conclusion, our study observed no significant difference between amenamevir and valacyclovir regarding the risk of zoster‐related hospitalization. Given its once‐daily dosing and lack of the need for renal dose adjustment, amenamevir may be a practical treatment option for herpes zoster, particularly in primary care settings, where renal function cannot be readily assessed. However, the possibility of increased ocular complications and limitations in the generalizability of our results should be considered.

## Funding

This study was supported by a grant from the Ministry of Health, Labor and Welfare of Japan (23AA2003).

## Ethics Statement

This study was approved by the Institutional Review Board of the University of Tokyo (2021010NI, April 23, 2021). The board waived the need for informed consent owing to the anonymous nature of the data source.

## Conflicts of Interest

The authors declare no conflicts of interest.

## Supporting information


**Table S1:** Emulated target trial framework for amenamevir vs. valacyclovir in patients with herpes zoster.
**Table S2:** Definitions of the inclusion and exclusion criteria.
**Table S3:** Definitions of outcomes.
**Table S4:** Definitions of the covariates.
**Table S5:** Sensitivity analysis using propensity score matching instead of overlap weighting.
**Table S6:** Sensitivity analysis using conventional stabilized inverse probability of treatment weighting instead of overlap weighting.
**Table S7:** Sensitivity analysis using an alternative definition that additionally required a herpes zoster‐related complication diagnostic code on the day of admission.
**Figure S1:** Study design diagram.

## Data Availability

The data used in this study were obtained from DeSC Healthcare Inc. under license and are not publicly available because of contractual and privacy restrictions. Access may be requested directly from DeSC Healthcare Inc., subject to its eligibility criteria and data use agreements.
