# Supplementary material for: Effectiveness of Amenamevir Vs. Valacyclovir in Preventing Herpes Zoster‐Related Hospitalization and Complications: A Target Trial Emulation Using a Large Japanese Claims Database
Source: J Dermatol. 2026 Jun 9;53(8):1115–23. doi: 10.1111/1346-8138.70349 (PMC13435107; doi:10.1111/1346-8138.70349)
Supplement: Supplementary file 1 [file JDE-53-1115-s001.docx]

**List of supplemental data**

**Table S1.** Emulated target trial framework for amenamevir versus valacyclovir in patients with herpes zoster

**Table S2**. Definitions of the inclusion and exclusion criteria

**Table S3**. Definitions of outcomes

**Table S4**. Definitions of the covariates

**Table S5**. Sensitivity analysis using propensity score matching instead of overlap weighting

**Table S6**. Sensitivity analysis using conventional stabilized inverse probability of treatment weighting instead of overlap weighting

**Table S7**. Sensitivity analysis using an alternative definition that additionally required a herpes zoster-related complication diagnostic code on the day of admission

**Figure S1**. Study design diagram

**Table S1.** Emulated target trial framework for amenamevir versus valacyclovir in patients with herpes zoster

| Protocol component | Target trial | Emulation |
| --- | --- | --- |
| Eligibility criteria | Inclusion criteria   1) Patients diagnosed with herpes zoster between September 7, 2017, and July 31, 2024, in the outpatient setting   Exclusion criteria   1) Age < 18 years 2) History of herpes zoster-related diseases 3) Previous use of amenamevir or valacyclovir 4) Hospitalization on the day of diagnosis 5) Coexistent varicella diagnosis 6) Complications due to herpes zoster, including encephalitis, meningitis, disseminated zoster, and ocular lesions present at diagnosis 7) Prescription of two or more, systemic (oral or intravenous), varicella-zoster virus-active antiviral drugs (amenamevir, valacyclovir, acyclovir, vidarabine, or famciclovir) on the day of diagnosis 8) Rifampicin use 9) Dialysis 10) Pregnancy 11) HIV infection | Inclusion criteria  Same as for the target trial.  1) Same as for the target trial  2) Initiation of amenamevir or valacyclovir  The additional criterion 2) was necessary for target trial emulation because, in real-world practice, some patients may not receive antiviral therapy or may receive antiviral drugs other than amenamevir or valacyclovir.  Exclusion criteria  1)–11) Same as for the target trial  12) Lacking ≥ 365 days of continuous enrollment in the database  13) Lacking data of baseline estimated glomerular filtration rate  Regarding the criterion 2), herpes zoster-related diseases were identified using the ICD-10 code “B02.x.” |
| Treatment strategies | Initiation of amenamevir or valacyclovir at randomization. Decisions on treatment duration were left to the clinician’s discretion | Same as the target trial |
| Assignment procedures | Participants randomly assigned to amenamevir or valacyclovir | Participants were classified according to the treatment initiated on the index date ( first prescription date of amenamevir or valacyclovir). Randomization was emulated by estimating propensity scores using logistic regression with prespecified baseline covariates and applying overlap weighting. |
| Follow-up period | The follow-up period starts at randomization and ends at the earliest occurrence of one of the following events: the study outcome of interest, death, loss to follow-up, 30 days after the index date, or the end of the study period (July 31, 2024). | Same as the target trial |
| Outcome | Primary outcome: Hospitalization due to acute zoster-related complications within 30 days.  Secondary outcomes: Zoster-related complications, including zoster encephalitis, zoster meningitis, disseminated zoster, zoster ocular disease, and acute kidney injury, within 30 days. | Primary outcome: Any hospitalization within 30 days after the index date with intravenous acyclovir or vidarabine administered on the day of admission or the following day.  Secondary outcomes: Same as those for the target trial. |
| Causal contrasts of interest | Intention-to-treat effect | Observational analog of intention-to-treat effect |
| Analysis plan | Intention-to-treat analysis: 30-day risk differences and risk ratios were calculated. | Same as the target trial |

Abbreviations: ICD-10, International Classification of Diseases, 10th Revision; HIV, human immunodeficiency virus

**Table S2.** Definitions of the inclusion and exclusion criteria

| **Inclusion criteria** | **Definition** | **Assessment period^a^** |
| --- | --- | --- |
| Herpes zoster | ICD-10 code (standard disease code): B02.9 or B02.2 (0531010: trigeminal herpes zoster) | [0, 0] |
| Initiate oral antiviral treatment with amenamevir or valacyclovir | ATC code: J05AX26 (amenamevir) or J05AB11  (valacyclovir) | [0, 0] |
| **Exclusion criteria** | **Definition** | **Assessment period^a^** |
| Age | < 18 years | [0, 0] |
| Inability to ensure a 365-day look-back period | Continuous enrollment (or observable data) < 365 days prior to the index date | [–365, 0] |
| Lacking data of baseline eGFR | Lacking medical checkup data, including eGFR | [–365, –1] |
| History of herpes zoster-related diseases | ICD-10 code: B02.x | [–365, –1] |
| Previous prescription of study drugs | ATC code: J05AX26 (amenamevir) or J05AB11 (valacyclovir) | [–365, –1] |
| Hospitalization at the index date | Hospitalization at the index date due to any reason | [0, 0] |
| Varicella present at diagnosis | ICD-10 code: B01.x | [0, 0] |
| Zoster encephalitis present at diagnosis | ICD-10 code: B02.0 | [0, 0] |
| Zoster meningitis present at diagnosis | ICD-10 code: B02.1 | [0, 0] |
| Disseminated zoster present at diagnosis | ICD-10 code: B02.7 | [0, 0] |
| Zoster ocular disease present at diagnosis | ICD-10 code: B02.3 | [0, 0] |
| Prescription of two or more, systemic (oral or intravenous), varicella-zoster virus-active antiviral drugs on the day of diagnosis | Varicella-zoster virus-active antiviral drugs are defined as follows:  ATC code: J05AX26 (amenamevir), J05AB11 (valacyclovir), J05AB01 (acyclovir), J05AB03 (vidarabine), and J05AB09 (famciclovir) | [0, 0] |
| Rifampicin use | ATC code: J04AB02 | [–90, 0] |
| Dialysis | Procedure code: J038 (hemodialysis), J042 (peritoneal dialysis) | [–90, 0] |
| Pregnancy | ICD-10 code: O00.x–O99.x, Z33.x–Z36.x | [–90, 0] |
| HIV infection | ICD-10 code: B20.x–B24.x | [–365, 0] |

ATC, Anatomical Therapeutic Chemical; ICD-10, International Classification of Diseases, 10th Revision; eGFR, estimated glomerular filtration rate; HIV, human immunodeficiency virus

^a^Follow-up started on the index date (day 0). We evaluated the inclusion and exclusion criteria during the assessment period.

**Table S3.** Definitions of outcomes

| Outcome | Definition | Assessment period^a^ |
| --- | --- | --- |
| Primary | | |
| Hospitalization for acute complications of herpes zoster | Any hospitalization within 30 days after the index date with intravenous acyclovir or vidarabine administered on the day of admission or the following day.  ATC code: J05AB01 (acyclovir), J05AB03 (vidarabine) | [1, 30] |
| Secondary | | |
| Zoster encephalitis | Diagnosis of zoster encephalitis within 30 days after the index date  ICD-10 code: B02.0 | [1, 30] |
| Zoster meningitis | Diagnosis of zoster meningitis within 30 days after the index date  ICD-10 code: B02.1 | [1, 30] |
| Disseminated zoster | Diagnosis of disseminated zoster within 30 days after the index date  ICD-10 code: B02.7 | [1, 30] |
| Zoster ocular disease | Diagnosis of zoster ocular disease within 30 days after the index date  ICD-10 code: B02.3 | [1, 30] |
| Acute kidney injury | Diagnosis of acute kidney injury or dialysis within 30 days after the index date  ICD-10 code: N17.x  Procedure code: J038 (hemodialysis), J042 (peritoneal dialysis) | [1, 30] |

Abbreviations: ATC, Anatomical Therapeutic Chemical; ICD-10, International Classification of Diseases, 10th Revision.

^a^Follow-up started on the index date (day 0). We evaluated the outcomes during the assessment period.

**Table S4.** Definitions of the covariates

| Characteristic | Definition | Assessment period^a^ |
| --- | --- | --- |
| Baseline eGFR | Following laboratory result retrieved from the most recent annual health checkup data. | [–365, –1] |
| Craniocervical distribution | ICD-10 code (standard disease code): B02.9 (0539002, 8833636, 8833404), B02.2 (0531010) | [0, 0] |
| Hypertension | ICD-10 code: I10, I11.x–I13.x, I15.x | [–365, –1] |
| Dyslipidemia | ICD-10 code: E78.x | [–365, –1] |
| Diabetes mellitus | ICD-10 code: E10.x–E14.x | [–365, –1] |
| COPD | ICD-10 code: J43.x, J44.x | [–365, –1] |
| Congestive heart failure | ICD-10 code: I50.x | [–365, –1] |
| Coronary artery disease | ICD-10 code: I20.x–I25.x | [–365, –1] |
| Stroke | ICD-10 code: I61.x, I63.x–I64.x | [–365, –1] |
| Immune system disorders | ICD-10 code: D80.x–84.x, D89.x | [–365, –1] |
| Solid tumor | ICD-10 code: C00.x–C80.x, D00.x–D09.x | [–365, –1] |
| Hematological malignancies | ICD-10 code: C81.x–C96.x | [–365, –1] |
| Other hematological diseases with immunocompromised conditions | ICD-10 code: D70, D61.x, D46.x | [–365, –1] |
| Organ transplants | ICD-10 code: Z94.x | [–365, –1] |
| Charlson Comorbidity Index | 0, 1, 2, ≥ 3 | [–365, –1] |
| NSAIDs | ATC code: M01A | [–365, –1] |
| Systemic glucocorticoids | ATC code: H02AB | [–365, –1] |
| Antineoplastic agents | ATC code: L01 | [–365, –1] |
| Immunosuppressants | ATC code: L04 | [–365, –1] |
| Treatment facility type | Clinic, hospital, or others (dental hospital or clinic) | [0, 0] |
| Healthcare utilization | Number of outpatient visits (≤ 11, 12–23, ≥ 24), hospitalization within the previous 365 days (yes/no) | [–365, –1] |
| Health insurance plan type | National Health Insurance (*Kokuho*), Health Insurance Societies (*Kempo*), or Advanced Elderly Medical Service System | [0, 0] |
| Fiscal year of index date | 2017–2024 | [0, 0] |

Abbreviations: ATC, Anatomical Therapeutic Chemical; COPD, chronic obstructive pulmonary disease; eGFR, estimated glomerular filtration rate; ICD-10, International Classification of Diseases, 10th Revision; NSAIDs, nonsteroidal anti-inflammatory drugs

^a^Follow-up started on the index date (day 0). We evaluated the covariates during the assessment periods.

**Table S5**. Sensitivity analysis using propensity score matching instead of overlap weighting

| Outcomes | Amenamevir | Valacyclovir | RR (95% CI) | RD (95% CI) |
| --- | --- | --- | --- | --- |
| **Primary outcome (%)** |  |  |  |  |
| 30-day zoster-related hospitalization | 0.75% (0.76%) | 0.74% (0.71%) | 1.02 (0.85–1.23) | 0.01% (–0.12%–0.15%) |
| **Secondary outcomes (%)** |  |  |  |  |
| Diagnosis of zoster encephalitis | 0.02% (0.02%) | 0.01% (0.01%) | 1.67 (0.40–6.97) | 0.01% (–0.01%–0.03%) |
| Diagnosis of zoster meningitis | 0.04% (0.04%) | 0.02% (0.02%) | 2.00 (0.75–5.33) | 0.02% (–0.01%–0.05%) |
| Diagnosis of disseminated zoster | 0.12% (0.13%) | 0.10% (0.09%) | 1.23 (0.76–2.00) | 0.02% (–0.03%–0.08%) |
| Diagnosis of zoster ocular disease | 0.83% (0.86%) | 0.64% (0.66%) | 1.29 (1.07–1.56) | 0.19% (0.05%–0.32%) |
| Diagnosis of AKI or initiation of dialysis | 0.02% (0.02%) | 0.09% (0.08%) | 0.22 (0.09–0.54) | –0.07% (–0.11% to –0.03%) |

Abbreviations: AKI, acute kidney injury; CI, confidence interval; RD, risk difference; RR, risk ratio

Values in parentheses represent crude outcomes. RR was calculated as the risk in the amenamevir group/risk in the valacyclovir group, and RD was calculated as the risk in the amenamevir group – risk in the valacyclovir group. RR > 1 and RD > 0 indicated a higher risk of amenamevir use.

**Table S6**. Sensitivity analysis using conventional stabilized inverse probability of treatment weighting instead of overlap weighting

| Outcomes | Amenamevir | Valacyclovir | RR (95% CI) | RD (95% CI) |
| --- | --- | --- | --- | --- |
| **Primary outcome (%)** |  |  |  |  |
| 30-day zoster-related hospitalization | 0.73% (0.76%) | 0.73% (0.71%) | 1.00 (0.84–1.20) | 0.00% (–0.13% to 0.13%) |
| **Secondary outcomes (%)** |  |  |  |  |
| Diagnosis of zoster encephalitis | 0.02% (0.02%) | 0.01% (0.01%) | 2.08 (0.62–6.98) | 0.01% (–0.01% to 0.03%) |
| Diagnosis of zoster meningitis | 0.04% (0.04%) | 0.02% (0.02%) | 2.42 (0.92–6.36) | 0.03% (–0.00% to 0.05%) |
| Diagnosis of disseminated zoster | 0.13% (0.13%) | 0.10% (0.09%) | 1.25 (0.79–1.97) | 0.03% (–0.03% to 0.08%) |
| Diagnosis of zoster ocular disease | 0.84% (0.86%) | 0.67% (0.66%) | 1.25 (1.05–1.49) | 0.17% (0.04% to 0.30%) |
| Diagnosis of AKI or initiation of dialysis | 0.02% (0.02%) | 0.08% (0.08%) | 0.23 (0.10–0.53) | –0.06% (–0.10% to –0.03%) |

Abbreviations: AKI, acute kidney injury; CI, confidence interval; RD, risk difference; RR, risk ratio.

Values in parentheses represent the crude outcomes. RR was calculated as the risk in the amenamevir group/risk in the valacyclovir group and RD as the risk in the amenamevir group – risk in the valacyclovir group. RR > 1 and RD > 0 indicate a higher risk of amenamevir use.

**Table S7**. Sensitivity analysis of the primary outcome using an alternative definition that additionally required an herpes zoster-related complication diagnostic code on the day of admission

| Outcomes | Amenamevir | Valacyclovir | RR (95% CI) | RD (95% CI) |
| --- | --- | --- | --- | --- |
| **Revised primary outcome (%)** | 0.18% (0.19%) | 0.16% (0.15%) | 1.12 (0.78–1.62) | 0.02% (–0.04%–0.08%) |

Abbreviations: CI, confidence interval; RD, risk difference; RR, risk ratio

Values in parentheses represent crude outcomes. RR was calculated as the risk in the amenamevir group/risk in the valacyclovir group, and RD was calculated as the risk in the amenamevir group – risk in the valacyclovir group. RR > 1 and RD > 0 indicate a higher risk of amenamevir use.

**Figure S1.** Study design diagram

^a^Zoster encephalitis, zoster meningitis, disseminated zoster, and zoster ocular disease at the time of diagnosis.

^b^Varicella-zoster virus-active antiviral drugs include amenamevir, valacyclovir, acyclovir, vidarabine, and famciclovir, administered orally or intravenously.

^c^Comorbidities included hypertension, dyslipidemia, diabetes mellitus, chronic obstructive pulmonary disease, congestive heart failure, coronary artery disease, stroke, immune system disorders, solid tumors, hematological malignancies, other hematological diseases with immunocompromised conditions, organ transplants, and Charlson Comorbidity Index.

^d^Healthcare utilization included the number of outpatient visits and hospitalization for any reason within the previous 365 days.

^e^Medication use included nonsteroidal anti-inflammatory drugs and immunosuppressive drugs (systemic glucocorticoids, antineoplastic agents, and immunosuppressants).

^f^Baseline characteristics included sex, age, treatment facility type, health insurance plan type, and index year.

^g^The earliest outcome of interest was death, loss of insurance eligibility (end of observable data), or 30 days of follow-up.
